# Two Cases of Mesonephric-Like Adenocarcinoma Arising From Ovary and Peritoneal and Literature Review

**DOI:** 10.7759/cureus.94394

**Published:** 2025-10-12

**Authors:** Hinako Sano, Yu Horibe, Atsuko Seki, Saeko Yoshizawa, Tsutomu Tabata

**Affiliations:** 1 Gynecology, Tokyo Women's Medical University, Tokyo, JPN; 2 Diagnostic Pathology, Tokyo Women's Medical University, Tokyo, JPN

**Keywords:** gynecologic oncology, immunohistochemistry, mesonephric-like adenocarcinoma, ovarian cancer, primary peritoneal carcinoma

## Abstract

Reports of Mesonephric-like adenocarcinoma (MLA) arising from other than the ovary and uterus are exceedingly rare. This report details two cases of MLA in patients with a history of endometriosis. One of these two cases is believed to be the first documented case of primary peritoneal MLA.

The first case was a 64-year-old woman with a history of hysterectomy and oophorectomy for endometriosis. She presented with International Federation of Gynecology and Obstetrics (FIGO) stage IVB primary peritoneal MLA, diagnosed eight years later via CT-guided biopsy and immunohistochemistry. Despite multiple chemotherapy regimens, she died 13 months after initiation of treatment. The second case was a 72-year-old woman with a history of endometriosis, and she was diagnosed with FIGO stage IIIA1(i) ovarian MLA. She underwent complete cytoreductive surgery followed by adjuvant paclitaxel and carboplatin chemotherapy and remained recurrence-free for three years. A literature review of 66 reported cases of ovarian MLA was conducted to compare clinical characteristics, treatment, and prognosis. According to these findings, 55% were diagnosed at an early stage (FIGO I-II), and prognosis was generally favorable after complete surgical resection, but outcomes were poor for sub-optimally resected cases.

These cases suggest a potential link between MLA development and a history of endometriosis, particularly after surgical intervention. The prognosis for MLA is highly dependent on achieving complete surgical resection. Advanced-stage primary peritoneal MLA has a poor prognosis, comparable to conventional primary peritoneal serous carcinoma. Given the difficulty of preoperative diagnosis, diligent follow-up is recommended for high-risk patients with a history of endometriosis. Future research is needed to establish effective treatments for advanced or recurrent disease.

## Introduction

Mesonephric-like adenocarcinoma (MLA) is a recently recognized histopathological entity, added in the 2020 World Health Organization classification [1]. Due to the limited number of reported cases, its clinical features and optimal treatment have not yet been established. MLA is histopathologically similar to mesonephric adenocarcinoma (MA). While MA of the uterine cervix is a well-recognized malignant neoplasm that is thought to arise from mesonephric remnants, MLA is widely believed to originate from Müllerian ducts or Müllerian lesions, for example, endometriosis, serous tumors, clear cell carcinoma, or endometrioid carcinoma, with most reported cases arising from the uterus or ovary [2-4]. MLA originating in other pelvic organs is extremely rare. MLA tumors exhibit a diverse range of architectural patterns, including tubular, pseudo-endometrioid, slit-like, papillary, and solid arrangements [5]. Periodic acid-Schiff-positive eosinophilic hyaline material is observed within the lumina. Immunohistochemically, PAX8, GATA3, thyroid transcription factor-1 (TTF-1), calretinin, and CD10 frequently show positive staining, whereas estrogen receptor (ER), progesterone receptor, and Wilms’ tumor 1 are typically negative [5].

Because MLA may resemble other pelvic neoplasms such as endometrioid, clear cell, or serous carcinoma, careful differential diagnosis is important [1]. For preoperative diagnosis, imaging modalities such as magnetic resonance imaging (MRI) may be employed. Although T2-weighted imaging (T2WI) often reveals uniformly low signal intensity, T1-weighted imaging (T1WI) demonstrates variable signal patterns. A definitive imaging-based diagnosis has yet to be established. Regarding treatment, when complete surgical resection is achieved, adjuvant chemotherapy with paclitaxel and carboplatin generally yields a favorable response. By contrast, cases in which only suboptimal or incomplete surgery is performed tend to have a poor prognosis.

We report two cases of MLA- one from the ovary, and the other is believed to be peritoneal primary. To our knowledge, there have been no prior reports of primary peritoneal MLA. We describe their clinical courses, diagnostic processes, and treatments, alongside a review of the relevant literature.

## Case presentation

Before presenting case one, we note that the cases included in this report were not consecutive but represent all patients diagnosed with ovarian MLA and presumed primary peritoneal MLA at our institution. These cases were encountered at Tokyo Women’s Medical University Hospital between February 2023 and May 2025. The inclusion was based on the rarity of this tumor entity, and all cases that met the histopathological criteria during this period were reported.

Case one

The patient was a 67-year-old G0 woman who was admitted to our hospital at the age of 48 years for evaluation of endometriotic cysts measuring 50 mm in the right ovary and 60 mm in the left ovary. She received six courses of leuprorelin acetate at a dose of 1.88 mg per injection. The cyst in the right ovary resolved, and the cyst in the left ovary was reduced to 31 mm. The patient was kept under observation. At the age of 59 years, the endometriotic cysts had enlarged to 60 mm in the left ovary and 41 mm in the right ovary. Pelvic contrast-enhanced MRI revealed a mural nodule with contrast enhancement within the left ovarian endometriotic cyst, raising suspicion of at least a borderline malignant tumor. A simple abdominal total hysterectomy and bilateral salpingo-oophorectomy were performed. The pathological diagnosis was an endometriotic cyst. The patient was discharged from our department the following year.

**Figure 1:**
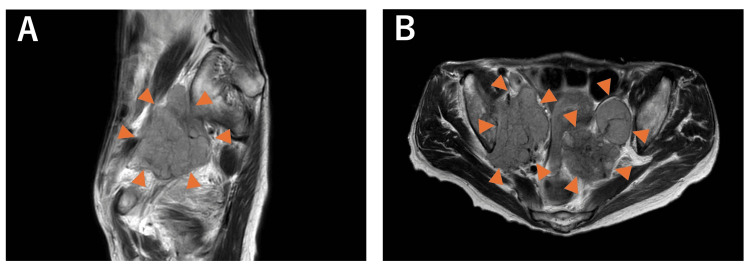
Pelvic contrast-enhanced MRI of case one Axial (A) and sagittal (B) T2WI images revealed a mass with intermediate signal intensity.

**Figure 2:**
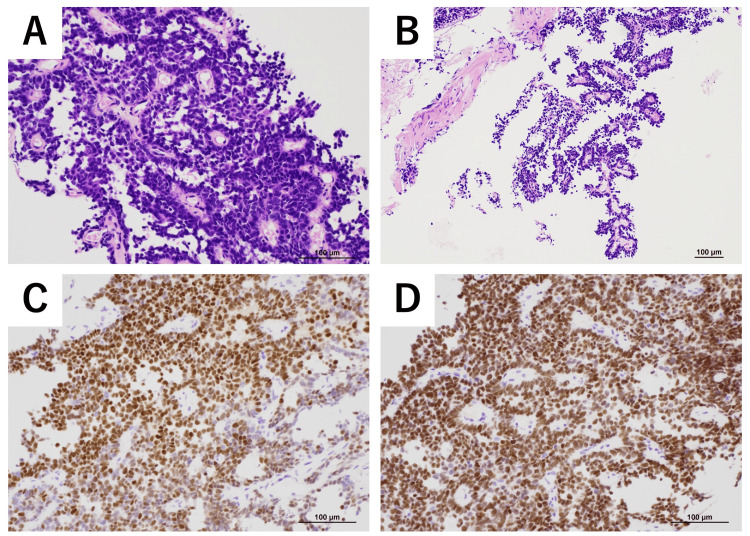
Histopathological and immunohistochemical images of case one (A) H&E staining, high-power view: gland-forming tumor with intraluminal eosinophilic material. (B) H&E staining, low-power view: papillary and infiltrative tumor fragments. (C) Immunohistochemistry for PAX8: diffuse nuclear positivity. (D) Immunohistochemistry for GATA3: diffuse nuclear positivity. (Scale bars: 100 µm.)

Eight years later, the patient developed fatigue, vomiting, and loss of appetite. Computed tomography (CT) performed at the previous hospital revealed a 60 mm mass in the posterior pelvic cavity close to the rectum, which was suspicious for malignancy, along with multiple liver and bone metastases, as well as enlarged lymph nodes along the right common iliac artery, bilateral internal and external iliac arteries, and near the rectum. Blood test results revealed elevated tumor marker levels, with a carcinoembryonic antigen (CEA) level of 3.4 ng/mL and a carbohydrate antigen 19-9 (CA19-9) level of 418 U/mL. Pelvic MRI showed a mass with intermediate signal intensity on both T1- and T2-weighted images, with restricted diffusion observed (Figure 1). A CT-guided biopsy of the pelvic tumor was performed. Histopathological examination revealed atypical cells with enlarged, hyperchromatic nuclei forming glandular or tubular structures, with eosinophilic hyaline material present within the lumina. Fibrovascular stroma and focal micropapillary proliferation were also observed (Figure 2). Immunohistochemical analysis of the same site showed positivity for PAX8 and GATA3, weak positivity for CD10, and negativity for calretinin, TTF-1, and ER (Figure 2). These findings were consistent with a diagnosis of MLA. On reevaluation of the uterus and bilateral adnexa that had been removed eight years earlier, only endometriotic tissue was identified, with no findings suggestive of MLA.

Given that eight years had elapsed since the hysterectomy and bilateral salpingo-oophorectomy, and considering that MLA arising from pelvic organs other than the uterus and ovaries is exceedingly rare, the lesion was presumed to be primary peritoneal in origin and was classified as International Federation of Gynecology and Obstetrics (FIGO) stage IVB. Two cycles of weekly paclitaxel at 175 mg/m² and carboplatin at an area under the curve (AUC) of 2 achieved a partial response. However, treatment was switched to irinotecan at 60 mg/m² and cisplatin at 60 mg/m² (50% dose) due to a drug-induced rash. After three cycles of this regimen, CT revealed new bone metastases, and the disease was classified as progressive disease. Therapy was then changed to weekly gemcitabine, which was administered for two cycles. However, because pulmonary metastases continued to enlarge, the treatment strategy was shifted to best supportive care. The patient died 13 months after the initiation of treatment.

Case two

**Figure 3:**
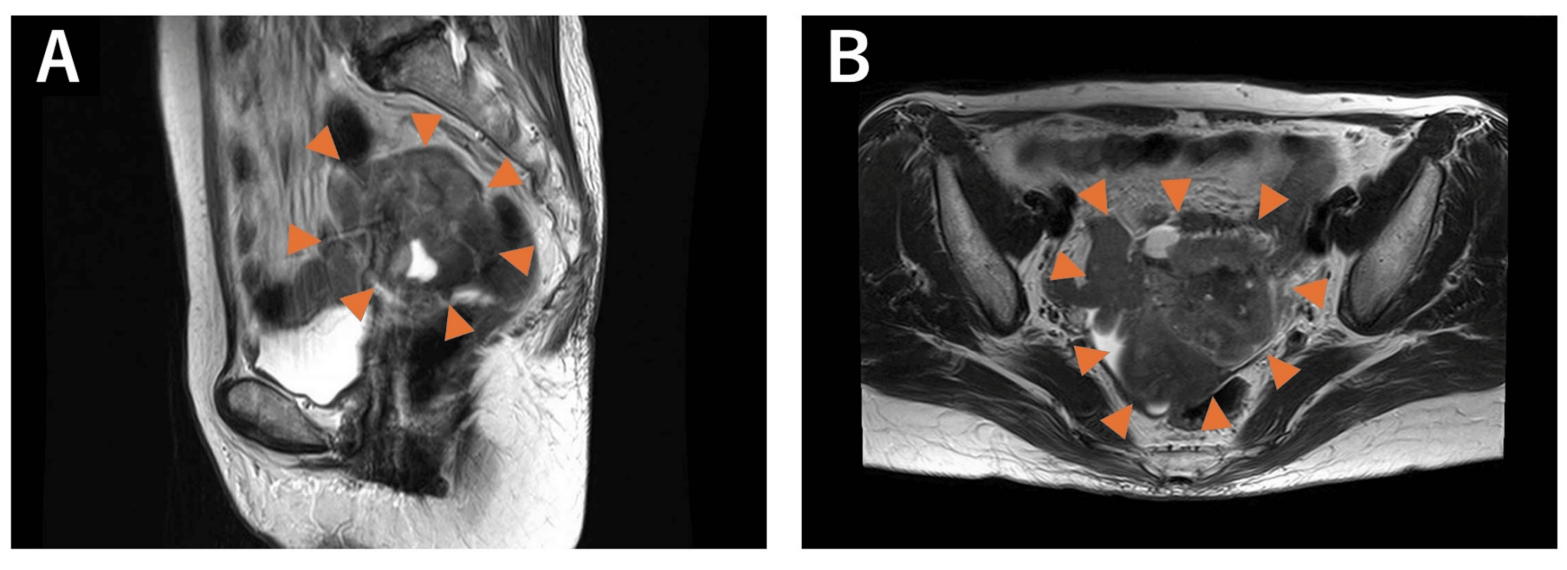
Pelvic contrast-enhanced MRI of case two Axial (A) and sagittal (B) T2WI revealed a mass in the left pelvis containing both solid and cystic components. The anterior part appeared solid, with mildly heterogeneous features and slightly high signal intensity on T2WI, whereas the posterior and medial parts appeared cystic, exhibiting low signal intensity on T2WI.

The patient is a 72-year-old woman, gravida 1, para 1, who presented to a previous hospital with vulvar pain, pruritus, and a 7 kg weight loss over six months. Transvaginal ultrasonography revealed a left ovarian mass, and she was referred to our hospital for further evaluation. MRI showed a left-sided pelvic mass with mixed solid and cystic components. The anterior portion appeared predominantly solid, with mildly heterogeneous signal intensity, faint hyperintensity on T2-weighted images, and peripheral-dominant diffusion restriction. In contrast, the posterior and medial portions appeared cystic and hemorrhagic, exhibiting high signal intensity on T1-weighted images and low signal intensity on T2-weighted images (Figure 3). CT demonstrated no obvious lymph node metastases. All tumor markers were within normal limits: CEA, 1.7 ng/mL; CA19-9, 26 U/mL; cancer antigen 125, 16 U/mL; squamous cell carcinoma antigen, 1.2 ng/mL; and alpha-fetoprotein, 3 ng/mL.

**Figure 4:**
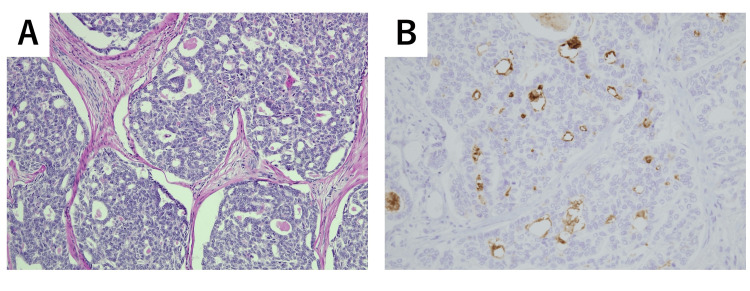
Histopathological and immunohistochemical images of case two (A) H&E staining: tubular and glandular architecture with intraluminal eosinophilic material. (B) Immunohistochemistry for CD10: focal weak positivity.

Based on the above findings, and under a clinical suspicion of left ovarian cancer, a simple total hysterectomy, bilateral salpingo-oophorectomy, pelvic and para-aortic lymph node dissection, and subtotal omentectomy were performed. Histopathological examination revealed atypical cells forming glandular and cribriform structures, with eosinophilic hyaline material present within the lumina (Figure 4). Immunohistochemical analysis of the same area showed positivity for PAX8 and GATA3, focal weak positivity for CD10, and negativity for calretinin, TTF-1, and ER, findings consistent with a diagnosis of MLA (Figure 4). According to the FIGO classification, the disease was staged as IIIA1(i). Six cycles of paclitaxel at 175 mg/m² and carboplatin at an AUC of 6 were administered as adjuvant chemotherapy. The patient has remained free of recurrence for three years postoperatively.

## Discussion

Pathogenesis

Several hypotheses have been proposed for the pathogenesis of MLA, the most widely accepted being that it arises from Müllerian ducts or Müllerian lesions, for example, Endometriosis, Serous tumors, Clear cell carcinoma, or Endometrioid carcinoma [2]. Other theories suggest that it may develop from endometriosis or even originate from germ cell tumors [3,6]. Reports of MLA arising from pelvic organs other than the uterus and ovaries are exceedingly rare. To date, no cases of primary peritoneal MLA have been documented in the literature. Additionally, two reported cases of MLA originating from the ureter and mesocolon shared several features in common: a history of endometriosis, prior surgical insult, and late onset, all of which were likewise observed in our present cases of peritoneal and ovarian MLA [3,6]. While not observed in the pathology of our cases, there is a report of pathological findings showing a close association and transition between endometriosis and MLA [3]. These findings support the hypothesis that MLA may arise from residual endometriotic tissue.

Diagnosis

We investigated the feasibility of preoperative diagnosis of MLA. A total of eight cases were compared: six previously reported cases that described the MRI features of ovarian MLA, along with the two present cases (Table 1). Although all cases exhibited a multilocular architecture with intermixed solid components, this is a non-specific feature that is also observed in common malignant ovarian tumors. Although some authors have proposed that malignant epithelial tumors exhibiting uniformly low signal intensity on T2-weighted images are characteristic [7], the wide range of signal intensities observed on T1-weighted images suggests that making a preoperative diagnosis based solely on imaging remains difficult.

**Table 1:** Imaging characteristics of MLA in eight cases Cases 1–7 were ovarian MLAs, and Case 8 was a peritoneal MLA. FIGO = International Federation of Gynecology and Obstetrics; T1WI = T1-weighted imaging; T2WI = T2-weighted imaging; DWI = diffusion-weighted imaging; Y = yes; N = no; NA = not available.

Cases	Study	FIGO stage	Complication	Multilocular	Solid component	T2WI signal	T1WI signal	Restricted diffusion	Contrast enhancement
1	Lee et al. [8]	IIA	Adult granulosa cell tumor	Y	Y	Low	Intermediate	NA	NA
2	Ujita et al. [7]	IC3	N	Y	Y	Low	Various	High on DWI	NA
3	Ishida et al. [4]	NA	Endometrioid adenofibroma	Y	NA	Moderate to low	High	High on DWI	NA
4	Yang et al. [9]	IIIA2	Mature cystic teratoma	NA	NA	Slightly high	Low	High on DWI	Uneven enhancement
5	Koh et al. [10]	IA (1), IC (3), II (1)	N	NA	Y	Low	NA	NA	NA
6	Sugitani et al. [11]	IC	N	Y	Y	Low	NA	Y	NA
7	Case one	IVB	Endometriosis	N	Y	Intermediate	Intermediate	Y	NA
8	Case two	IIIA1(i)	Endometriosis	Y	Y	Low	Low	Y	NA

With respect to tumor markers, only one case, reported by Kulkarni et al., showed an elevation of human epididymis protein 4, and its clinical utility has not yet been evaluated [12]. In addition, there have been reports of MLAs associated with endometriosis, serous borderline tumors, and endometrioid adenofibromas [1,4,9,13], suggesting that these lesions may serve as precancerous conditions. Cases with such associated conditions may be identifiable preoperatively. However, when evaluating biopsy or resection specimens, it can be difficult to differentiate MLA from endometrioid carcinoma; therefore, diagnosis must rely on both morphological and immunohistochemical characteristics. Given the diagnostic complexity of MLA, clinicians must remain alert to the risk of misdiagnosis, which could result in either overtreatment or undertreatment. Gynecologists and pathologists are therefore encouraged to collaborate proactively when encountering clinically or pathologically non-specific findings and to pursue further evaluation using the immunohistochemical markers described above [14].

Treatment/prognosis

Table 2 summarizes 66 reported cases of ovarian MLA, including the present case. Thirty-five of these 66 cases (53.0%) were classified as early-stage disease (FIGO stage I-II), while 14 cases (21.2%) were in the advanced stage. Among the 17 of 66 patients (25.8%) who presented with symptoms, 11 (64.7%) were in the early stage and 6 (35.3%) were in the advanced stage. Among the 29 of 66 cases (43.9%) with complications, 20 (69.0%) were in the early stage and 9 (31.0%) in the advanced stage. Of the 16 patients who underwent complete surgery, 12 (75.0%) were in the early stage and 4 (25.0%) were in the advanced stage.

**Table 2:** Clinical characteristics of 66 cases of ovarian MLA Y = yes; N = no; NA = not available; NED = no evidence of disease; AWD = alive with disease; PFS = progression-free survival; OS = overall survival; DSS = disease-specific survival; FU = follow-up; TC = paclitaxel + carboplatin; Bev = bevacizumab; BEP= Bleomycin + Etoposide + Cisplatin; ATH = abdominal total hysterectomy; BSO = bilateral salpingo-oophorectomy; OMT = omentectomy; pOMT = partial omentectomy; LND = lymphadenectomy; PLND = pelvic lymphadenectomy; CRS = cytoreductive surgery; PALND = para-aortic lymphadenectomy; LSO = left salpingo-oophorectomy.

Cases	Study	Age	Symptom	Complication	FIGO stage	Treatment	Chemotherapy	Recurrence	Follow- up
1-5	McFarland et al. [15]	42-72 (mean=60)	NA	Endometriosis (3), none (2)	IA(3), IB(1), IIIC(1)	NA	NA	N (IA, IB), Y(IIIC)	18 (IA, IB), 12 (IIIC)
										6	Chapel et al. [16]	80	Abdominal pain	Serous borderline tumor and low-grade serous carcinoma	Ⅳ	ATH+BSO+OMT+CRS	TC	N	3 NED
7	McCluggage et al. [5]	61	Pelvic pain, discharge, mass	Serous borderline tumor)	IIIA1	ATH+BSO+Peritonectomy+OMT+LND+CRS	TC	NA	NA
8	McCluggage et al. [5]	66	NA	Borderline endometrioid adenofibroma	NA	NA	NA	NA	NA
9	McCluggage et al. [5]	77	NA	Endometriosis; mixed serous and mucinous cystadenoma	NA	NA	NA	NA	NA
10	McCluggage et al. [5]	50	NA	none	NA	NA	NA	NA	NA
11	McCluggage et al. [5]	73	NA	Serous cystadenoma	NA	NA	NA	NA	NA
12	Pors et al. [17]	67	NA	none	IC	NA	NA	NA	NA
13-15	Kezlarian et al. [18]	36-45	NA	none (3)	III(1), IIIA(1), NA(1)	NA	NA	NA	NA
16	Dundr et al. [13]	61	NA	Serous borderline tumor	Ⅳ	ATH+BSO+Liver/Diaphragm/OMT/Appendectomy/Umbilical Met Resection	TC＋Bev	N	12 NED
17	Seay et al. [2]	67	Heaviness, polyuria	Endometriosis	IA	ATH+PLND+Infracolic OMT+Adhesiolysis	TC+Bev	Y	18 months
18	Chen et al. [19]	29	Abdominal discomfort	Serous borderline tumor	IC2	ATH+BSO	TC	N	13 months
19	Ujita et al. [7]	84	N	none	IC	ATH+BSO	N	N	4 NED
20	Kulkami et al. [12]	67	Nausea, bloating, weight loss	Endometriosis	IIIA	ATH+BSO+OMT+LND biopsy	TC+Bev	Y(lung)	AWD
21-25	Koh et al. [10]	42-67	Pelvic mass, Distension, none	Endometriosis (1), Endometriotic cyst (3), none (1)	IA(1), IC(3), IIB(1)	Various	TC(4), N(1)	Y(2), N(3)	NA
26	Ishida et al. [4]	69	Lower abdominal pain	Endometrioid adenofibroma	IIB	ATH＋BSO	TC	lung	8 AWD
27	Xu et al. [20]	78	N	Endometrioid adenofibroma	Ⅰ	ATH+BSO+OMT+LND	NA	Y	NA
28	Lee et al. [8]	63	Abdominal discomfort	Adult granulosa cell tumors	IIA	ATH+BSO+OMT+LND	Y	NA	NA
29	Yang et al. [9]	45	Abdominal pain, mass	Endometriosis	IIB	LSO+PLND+LND+Small Bowel/Peritoneal Resection	TC	N	4 months
30-54	Pors et al. [1]	36–81 (mean=61)	NA	NA	I(13), II–IV(7), none(5)	NA	NA	Y (: lungs, liver, etc.)	1–1346 months (mean 101), 5-year PFS 68%, OS/DSS 71%
55	Nilforoushan et al. [21]	58	NA	Serous borderline tumors	IVB	NA	NA	NA	NA
56	Nilforoushan et al. [21]	70	NA	Serous borderline tumor	IVB	NA	NA	NA	NA
57	Zhang et al. [9]	67	Pelvic mass, urination	Seromucinous neoplasm, endometriosis	IC	ATH+BSO+OMT+Appendectomy+PLND	TC	NA	4 months
58	Fan et al. [9]	66	Nausea, vomiting, distention	none	IB	ATH+BSO+OMT+Adhesiolysis	NA	N	12 months
59	Zhang et al. [9]	61	Abdominal distention	none	IVB	CRS	TC	N	13 months
60	Deolet et al. [6]	33	NA	mature teratoma	IA	LSO	Carboplatin+Gemcitabine	Y	46 months (19 months NED)
61	Deolet et al. [6]	75	NA	High grade serous carcinoma	IIIC	ATH+BSO+OMT	TC+Bev	N	22 NED
62	Deolet et al. [6]	68	NA	Borderline endometrioid neoplasm	IC	BSO	N	N	28 NED
63	Deolet et al. [6]	49	NA	mixed germ cell tumor	IVB	Right Partial Oophorectomy	BEP	Partial response	8 months partial response
64	Kim et al. [22]	47	Pelvic mass	NA	IIIC	PLND+PALND+OMT+Peritonectomy	NA	N	11 NED
65	Sugitani et al. [11]	53	N	Serous cystadenoma	IC1	ATH+BSO+pOMT	TC	N	NA
66	Current	72	Weight loss	Endometriosis	IIIA1(i)	ATH+BSO+OMT+LND+Adhesiolysis	TC	N	24 NED

Generally, 70-80% of epithelial ovarian cancer is diagnosed at an advanced stage. The five-year survival rate is high-ranging from 70% to 89%-when detected early, but drops to 18-30% in advanced-stage disease, reflecting its poor prognosis [23,24]. Although no reports of peritoneal MLA have been published to date, and its prognosis remains unclear, the present case had a poor outcome comparable to that of conventional primary peritoneal serous carcinoma, which has a five-year overall survival rate of 30.6% [25]. In contrast, ovarian MLA can be diagnosed at an early stage in many cases (53.0%, 35 of 66 cases). Among these, 11 cases (31.4%) presented with symptoms-most commonly abdominal, which may have facilitated early diagnosis. However, no studies have specifically addressed the role of symptoms in diagnosis. Furthermore, patients tend to be older at onset (mean age, 61 ± 9.9 years), and coexisting lesions suggestive of precursor conditions, such as endometriosis, are common (43.9%, 29 of 66 cases). Following complete cytoreductive surgery, three of 10 patients (30%) who received adjuvant paclitaxel and carboplatin (TC) chemotherapy-administered according to standard ovarian cancer protocols-experienced recurrence. In the present case as well, achieving complete cytoreductive surgery is expected to contribute to a recurrence-free outcome, whereas patients who undergo suboptimal surgery or less tend to have a significantly poorer prognosis.

Treatment strategies following recurrence remain unclear. Because the positivity rates for homologous recombination deficiency and mismatch repair deficiency are low, some consider immune checkpoint inhibitors (ICIs) and poly ADP-ribose polymerase (PARP) inhibitors to have limited therapeutic value [11]; however, no definitive evidence in the literature currently supports this view. Based on two cases that may have arisen from endometriosis and followed the rare course of late malignant transformation, treating such patients in the same manner as those with conventional ovarian or peritoneal cancer may lead to a delay in accurate diagnosis, whether due to treatment initiated before diagnosis or to treatment based on misdiagnosis, and may ultimately worsen prognosis. Patients with residual endometriosis, therefore, warrant close surveillance. Even in advanced-stage disease-particularly in primary peritoneal MLA, the rapid progression and decline in quality of life suggest that a thorough genetic evaluation, including oncogene panel testing, is recommended.

## Conclusions

We report two cases of late-onset MLA in patients with a history of endometriosis, one of which represents, to our knowledge, the first documented case of likely primary peritoneal MLA. In our case, the prognosis of advanced-stage peritoneal MLA was poor. Early awareness of symptoms, past or present endometriosis, and prior intra-abdominal surgical trauma may be considered risk factors for the development of MLA; therefore, diligent follow-up may be advisable. Although no established non-invasive preoperative diagnostic methods-such as MRI assessment or tumor marker evaluation, are currently available, monitoring these parameters in conjunction with the patient’s subjective symptoms may help improve prognosis. Conversely, prognosis tends to remain poor in cases where complete surgical resection is difficult. In the future, in addition to chemotherapy and Poly(ADP-ribose) polymerase inhibitor (PARPi), we anticipate further reports on the potential usefulness of ICIs and antibody-drug conjugates, which show promise in the treatment of ovarian cancer.
